# Autoantibodies targeting neuronal proteins as biomarkers for neurodegenerative diseases

**DOI:** 10.7150/thno.72126

**Published:** 2022-03-28

**Authors:** Gabriela Kocurova, Jan Ricny, Saak V. Ovsepian

**Affiliations:** 1 Experimental Neurobiology Program, National Institute of Mental Health, Klecany, Czech Republic.; 2 Faculty of Science and Engineering, University of Greenwich London, Chatham Maritime, Kent, ME4 4TB, United Kingdom.

**Keywords:** Fluid biomarkers, autoimmunity, dementia, differential diagnosis, immunoglobins

## Abstract

Neurodegenerative diseases (NDDs) are associated with the accumulation of a range of misfolded proteins across the central nervous system and related autoimmune responses, including the generation of antibodies and the activation of immune cells. Both innate and adaptive immunity become mobilized, leading to cellular and humoral effects. The role of humoral immunity in disease onset and progression remains to be elucidated with rising evidence suggestive of positive (protection, repair) and negative (injury, toxicity) outcomes. In this study, we review advances in research of neuron-targeting autoantibodies in the most prevalent NDDs. We discuss their biological origin, molecular diversity and changes in the course of diseases, consider their relevance to the initiation and progression of pathology as well as diagnostic and prognostic significance. It is suggested that the emerging autoimmune aspects of NDDs not only could facilitate the early detection but also might help to elucidate previously unknown facets of pathobiology with relevance to the development of precision medicine.

## Introduction

Neurodegenerative diseases (NDDs) are chronic incurable disorders of the Central Nervous System (CNS) characterized by a progressive decline of synaptic functions and irreversible neuronal loss, with devastating personal impact and overwhelming socio-economical costs. With aging as the main risk factor, the most prevalent NDDs such as Alzheimer's disease (AD), Parkinson's disease (PD), Dementia with Lewy bodies (DLB), Frontotemporal Lobar Neurodegeneration (FTLD), Amyotrophic Lateral Sclerosis (ALS), and Vascular Dementia (VD) are currently on the rise 1, 2. Despite the considerable symptomatic overlap, NDDs are viewed as independent entities affecting specific functional systems of the CNS and manifesting via a set of distinctive symptoms and histopathological characteristics 3-7.

Amongst shared features of NDDs, deposition of misfolded proteins and fragments across CNS, neuroinflammation, dysregulation of glutamatergic signaling, oxidative stress with cytotoxic effects are the most prominent, contributing to neurological and psychiatric symptoms with behavioral impairments. Disruption of neuronal activity, synaptic transmission, and plasticity mechanisms are thought to be caused primarily by the accumulation of aggregation-prone toxic amyloid proteins in the CNS and dysregulation of Ca^2+^ homeostasis 8-13. Due to the alleged causal role and differential prevalence in various NDDs, amyloid proteins and their fragments accumulating in the brain and cerebrospinal fluid (CSF) prompted much interest as biomarkers for diagnosis, patient stratification, and monitoring the disease progression 14-18. The routine use of CNS tissue and CSF-based assays, however, is hampered by invasive procedures they involve with significant related health risks.

**Figure 1:**
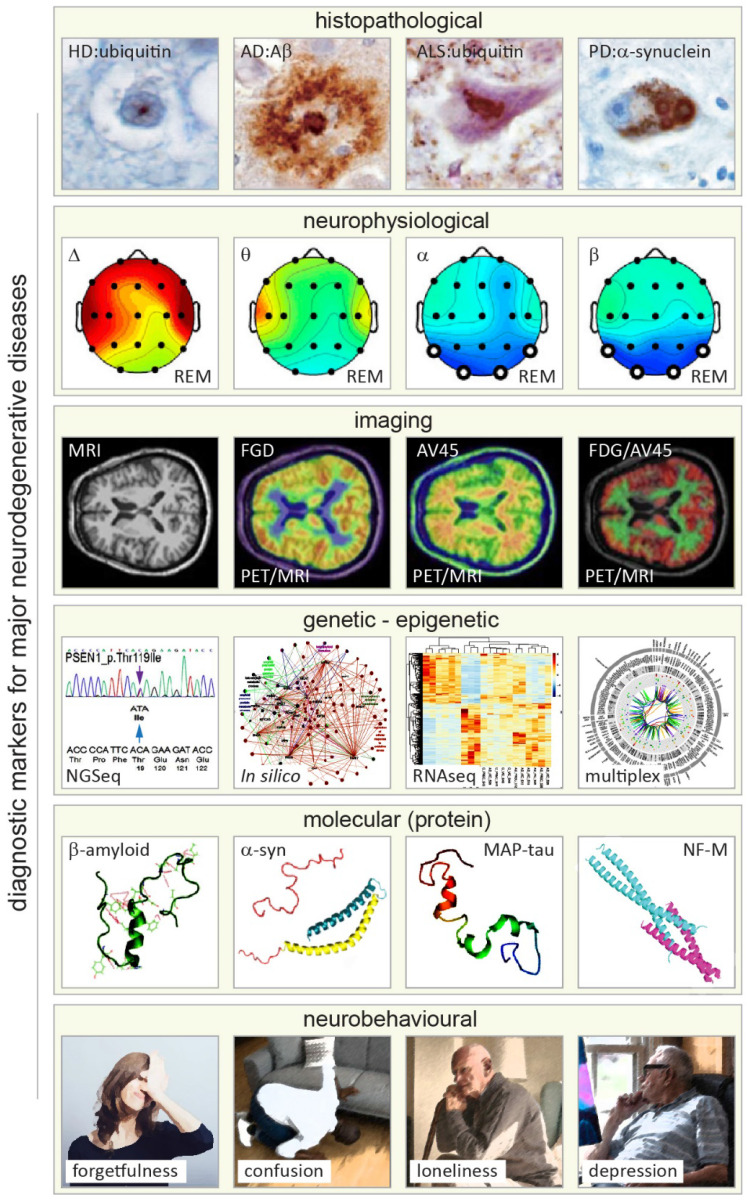
** Primary approaches and readouts used for diagnosis of neurodegenerative diseases (NDDs).** From top to bottom. First row: histopathological hallmarks of Huntington's, Alzheimer's, Lou Gehring's (known also as amyotrophic lateral sclerosis, ALS), and Parkinson's diseases shown in brain autopsy staining exemplifying deposition of distinguishing amyloid proteins (amyloid lesions, left to right). Adapted with permission from 142. Second row: neurophysiological readouts (electroencephalographic (EEG) maps) illustrating the distribution of neural dynamics and activity across various brain structures and areas in Alzheimer's disease with reference to changes in four major types of EEG activity (Δ, θ, α and β bands) in rapid eye movement (REM) phase of sleep (left to right). Adapted with permission from 143. Third row: magnetic and nuclear brain imaging (magnetic resonance imaging, MRI and positron emission tomography, PET) with various contrasts for detecting NDD-related changes in metabolic activity of the brain (Fluorodeoxyglucose, FDG) and amyloid distribution (Florpiramine F18; AV-45) targeting amyloid plaques, and hybrid MIR/PET, and dual FDG/AV-45 PET imaging modes (left to right). Adapted with permission from 144. Fourth row: primary genomic, transcriptomic, and bioinformatics (*in silico*) methods applied for diagnosis of NDDs analyzing genetic and epigenetic alterations (left to right). Adapted with permission from 145-147. Fifth row: 3D structure of four principal neuronal proteins enriched in amyloid deposits of the most prevalent NDDs (left to right). Note that for illustration purposes, the Ca^2+^ binding C-terminal domain of a-synuclein is truncated (pink). Adapted with permission from 148-151. Sixth row: major neurobehavioral symptoms of NDDs (exemplified by symptoms of Alzheimer's disease), which can vary between NDD conditions (Illustrations modified from iflScience.com).

Currently, there is a major unmet need for low-cost, non-invasive, and reliable methods for the early detection of CNS diseases. With advances in sensing technologies, it is expected that new approaches will be developed to facilitate the accurate diagnosis of NDDs and timely interventions 17, 19, 20 (Fig. 1). Over recent years, autoantibodies (Aabs) have generated much interest as putative biomarkers for NDDs 21-23. The abundance of Aabs in CSF and blood with their specific reaction to a range of neuronal proteins have been explicitly shown in preclinical studies as well as clinical reports involving patients 24-27. As emerges from this review, while major progress has been made in the analysis and characterization of Aab response in NDDs, the field is far from maturity, with numerous outstanding issues impeding the effective translation of Aabs-based approaches in diagnostic laboratories and clinical practice.

## Biology of Aabs with relevance to NDDs

Antibodies (Ab) are large Y-shaped proteins used by the immune system for recognizing and neutralizing foreign materials, through activating the complement system and phagocytosis. Abs are generated by two types of B lymphocytes: B1 and B2. B2 cells produce Abs in follicles of secondary lymphatic organs, which in their majority are regular proteins derived after specific antigenic stimulation 28. Some of these Abs may be directed against “auto” antigens, including those released from damaged and degenerating cells 29, 30. Up until now, the role of Aabs produced by B2 cells remained unclear, with emerging data suggesting their homeostatic effects. Unlike, Abs generated by B1 cells are typically poly-reactive and can be produced in the absence of extrinsic antigens (i.e., bacteria, viruses, fungi) or self-antigens. The latter account for ~5% of the whole Ab pool of blood. Because of their broad reactivity, Abs of B1 cells play a key role in wide-ranging first-line defense against infections and foreign proteins. For the same reason, a small portion of these Abs could demonstrate auto-reactivity, i.e. qualify as natural Aabs 31. In this way, B1 cells can play an important role in the clearance of cellular debris and removal of apoptotic tissue, protecting host organisms from toxic waste.

As natural immunoglobulins (Ig), Aabs occur in three isotypes: IgM, IgG, and IgA. IgM recognizes and binds post-apoptotic antigens and markers of cell senescence 32-34. Although IgM is mainly produced by CD5+ B1 cells, in limited amounts it can be also generated by B2 cells 35. Of all three immunoglobulins, IgM is the most abundant and of the lowest-affinity, whereas the amount of IgG and IgA are lower and vary considerably, with both showing higher immunoreactivity and specificity, as compared to IgM 36. It is noteworthy that IgM producing B2 cells respond poorly to receptor-mediated activation and rarely undergo affinity maturation. They may, however, undergo a class switching to generate high-affinity pathogenic IgG 37. Despite constant negative selection or targeted inactivation of self-reactive B lymphocytes in bone marrow, their positive selection can also occur. This process may lead to the emergence of immune cells producing Ab reacting to surface proteins of intact neurons and other brain cells, as well as peptides and proteins released after their pathological breakdown 38-40. Such autoimmune reaction has been implicated in autoimmune response-related psychosis and schizophrenia 41, 42 as well as neural autoantibody-associated dementias (NABD) with signs of axonal degeneration 43, 44. Quantitative analysis and profiling of Aabs targeting neuronal proteins, may, therefore, provide specific and instructive information on the onset, mechanisms, and severity of brain pathology.

## Exchange of immunoglobulins between peripheral circulation and CNS

CNS is considered immunologically privileged with very limited exposure to antigens and restricted infiltration of Abs taking place under physiological conditions. This is due to physical and molecular barriers at the blood-CNS interface (known as blood-brain barriers, BBB) and elaborate system of meningeal lymphatic vessels (mLVs) which control the concentration and isoforms of Abs entering the CNS and guide immune cells out to cervical lymph nodes 45. The latter is known to be the main site for presenting neuronal antigens to B lymphocytes and stimulating Ab production 46, 47. Nonetheless, there is growing evidence for a quantitative correlation of Aabs of the CFS and blood (serum), with their concentration in the CSF significantly lower than that in the peripheral circulation 48, 49. These findings suggest that under physiological conditions, a limited quantity of immunoglobulins can infiltrate the CNS from the peripheral circulation.

Many disorders affecting CNS, including NDDs, are associated with the disintegration of BBB, which may lead to an out-of-control outflow of neuronal and glial proteins with activation of autoimmune response 50, 51. Accordingly, a variety of Aabs target neuronal and glial proteins, and their changes can be detected in the blood and CSF of patients with NDDs. Amongst these, Aabs specific to neurofilament heavy subunit, tubulin, glial fibrillary acidic protein, S100b protein, tau, β-amyloid peptide, α-synuclein, myelin basic protein (MBP), and heparan sulfate proteoglycan are most extensively studied 52-56. Despite low amounts of Aabs in CSF, considerable evidence suggests their biological effects. In AD, for instance, Aabs may play dual, pathogenic, and protective roles, with levels of Ig recognizing self-antigens (protein tau, Aβ-amyloid peptide) correlating with specific disease stages and associated comorbidities.

## Amyloid-β Aabs

Gaskin et al. presented the first evidence for Aβ Aabs in the peripheral circulation of AD patients 57. This was followed by reports of Ig in serum of healthy and AD patients 58-65 in free form as well as in complex with Aβ, with complexation affecting the sensitivity of detection methods. In studies with dissociation of Aβ-Ab complex, the amount of detected Aβ was less variable 66. Most of Aβ42 and Aβ40 Aab studies showed a lower titer of unbound Ig in sera of AD patients as compared to healthy controls 59, 64, 67-69, with some reports also showing no difference 58 (Table 1). The lower levels of Aβ Aabs in serum of AD suggest the reduced passage of Aβ to blood, which could accelerate its accumulation in CNS and development of fibrillary deposits. Using ELISA for monomeric Aβ42 and aggregated soluble Aβ, Nath et al. found that titers of Aβ42 Aabs in serum of AD patients are higher as compared to patients with multiple sclerosis (MS) and HIV encephalitis. Comparative analysis showed that a significant fraction of Aβ Aabs in redox-treated serum peptides of clinical AD was reactive to Aβ oligomers, which were also reduced as compared to that in age-matched healthy controls. This observation infers that Aβ oligomer can leak from the CNS to plasma, supporting the potential usefulness of Aβ oligomer immunotherapy 68. Of note, in AD, the titre of monomeric Aβ1-42 Aabs in serum was lower than that for aggregated Aβ1-42, a finding implying that the immune response to Aβ targets specific conformational epitopes, which have higher immunogenicity in Aβ aggregates 63. Another report comparing serum IgG against Aβ1-42 mono- and oligomers in AD, MCI, and cognitively normal controls (10/group) with subtraction of polyvalent antibodies binding and dissociating Ab-Ig complexes did not find differences between the three groups 61. In contrast, analysis of the level of Aabs reactive to Aβ25-35 oligomers in serum showed their increase in AD patients as compared to controls 70. This short peptide is regarded as the main toxic domain of Aβ 71. Interestingly, longitudinal studies of Aabs changes in AD showed that the levels of Aabs to aggregated Aβ variants in sera increase during the mild to moderate phase of the disease but decline with the progression of the pathology into the severe phase 70.

Using an affinity purification approach, Mruthinti et al. found a higher titer of IgG binding Aβ42 peptide in plasma of AD 62. This observation agrees with the results of the earlier report with the use of acidic dissociation to measure bound and unbound antibodies, showing that their levels in AD exceeded that of age-matched controls 66, 72. It is important to note that exposure to low pH can cause partial denaturation of Aabs which can lead to increased reactivity 73. Other tests using Aabs specific to Aβ (21-37), and monoclonal mouse 6E10 antibody (mAb 6E10) that binds to Aβ (3-8) were also able to detect Aβ-IgG complexes in serum and CSF, which were more prevalent in AD patients. In combined analytical assays with clinical tests in AD patients, the titer of immune complexes in CSF and serum negatively correlated with the cognitive performance of subjects 74. A recent random-effect meta-analysis containing 30 case-control studies with a total of 2901 individuals (1311 and 1590, AD and healthy subjects, respectively) demonstrated an increase of Aβ IgG in the blood of AD, whereas IgM was lower in these subjects as compared to healthy. In the same report, assessments of CSF Aβ Aabs in AD against healthy showed no difference, while meta-regression analyses suggestive of measurable sex-related effects 75. Overall, although many studies advocate the diagnostic relevance of Aβ Aabs changes in the CSF and serum of AD, the results are controversial, calling for further research with careful stratification of subjects and the use of standardized methods.

## Microtubule protein tau Aabs

Microtubule-associated protein tau, which in AD becomes hyperphosphorylated (p-tau), is the main constituent of neurofibrillary tangles. An increase in the level of p-tau in the brain and CSF has been considered as one of the key biomarkers of AD 76-78. The presence of tau-reactive IgG and IgM have been reported in CSF and sera of AD patients, as well as in healthy controls 52, 53, 79-83 (Table 2). Tau Aabs were shown in various immunoglobulin (IVIG) products from large cohorts of healthy donors 81, 84-86 as well as in children 82 suggesting that they are unlikely to be harmful 82, 84 and may have some physiological role. Bartos and co-workers observed lower levels of tau-reactive Aabs in serum of AD patients as compared to controls, with titers declining further with the progression of the pathology 52. Considerable evidence suggests that anti-tau antibodies can infiltrate the CNS through impaired BBB to bind neurofibrillary tangles 87, 88 as well as intracellular tau deposits. The intracellular interaction may happen similar to the binding of paraneoplastic Ab to nuclear or cytoplasmic elements 89, 90. This process may interfere with the cytoskeletal functions, aggravating the disease process. It is interesting to note that reduction of tau Aabs in serum was also reported in PD 91. On the other hand, Rosenmann et al. have reported higher levels of IgM class Abs against p-tau in AD 83. This was, however, a pilot study using low number of samples, hence, the results warrant independent verification. Klaver et al. tested the binding of IgG and IgM from AD, MCI, and control subjects to p-tau and tau, using as antigens 196-207 tau peptide, as well as full-length variants (tau and p-tau at Serine-199 and Serine-202). Authors found specific antibodies to both p-tau and tau in most subjects, regardless of cognitive status, with increased specific IgG binding to p-tau (an increase in the p-tau IgG ratio) detected in MCI subjects as compared to AD patients and healthy controls 80.

Using circulating IgGs, it was shown that they can recognize modified tau variants, which differ in their characteristics 81, 85. These observations suggest that despite modifications of tau protein by aggregation, formation of paired helical filaments (PHFs), phosphorylation, and polymerization 92-96, they are still recognized by specific Aabs. Preliminary data from our laboratory (PI, Dr. Ricny) showed that tau Ab from the serum of AD patients interact equally with both, recombinant and natural monomeric tau derived from brain homogenates 97. On the contrary, antibodies isolated from IVIG and pooled from the plasma of healthy controls showed stronger reactivity with recombinant tau fragment (155-421 aa) and with aggregated forms 81, 85. Notwithstanding considerable research, currently, there is a lack of consensus if tau Aabs levels are altered in the peripheral circulation of AD and MCI patients. It is important to note that the results of clinical trials with anti-Aβ antibodies leading to the removal of amyloid plaques suggest that neurofibrillary tangle pathology is secondary to the build-up of amyloid deposits, and the reversal of tau pathology might be important in the onset of clinical benefits with cognitive improvements 98. Currently, it is unclear if anti-tau Aabs have any protective role or can influence the formation of NF tangles, which in turn could influence the spread of tau pathology and cognitive functions.

## Neurofilament Aabs

Neurofilaments (NFs) belong to a family of intermediate filaments with their diameter (~10 nm) falling between two other cytoskeletal polymers, i.e., microtubules (~25 nm) and actin (~6 nm). Based on their gene sequence and structural characteristics, NFs are divided into six types (I-VI) 99, 100. Adult neurons in CNS are enriched with pan-neuronal type IV NFs (i.e. NF triplet proteins light, middle and heavy, NF-L, NF-M, NF-H, and α-internexin), while peripheral neurons express NF triplet proteins with type III IF peripherin 101, 102. NFs are integral structural elements of synapses, enriched especially at postsynaptic sites of glutamatergic synapses, with their impairments disrupting synaptic plasticity and memory formation in animal models, and implicated in several NDDs and neuropsychiatric conditions 103.

NF deposits were found to co-localize with tau tangles in brains affected by AD 104 as well as within LBs of dopaminergic cells in PD 105 and dystrophic neurites of ALS motor neurons 106. Increased levels of NF in the blood and CSF infers axonal injury, which can result as a part of normal brain aging and pathological processes, such as autoimmune diseases, inflammation, vascular and traumatic disorders of the CNS and PNS 101, 107, 108 (Table 2). Higher NF levels in peripheral circulation have been reported in association with neuronal damage caused by several NDDs 17, 109-111. Although the ubiquitous presence of NFs in the CNS and their release in CSF and blood in various NDDs rule out their utility for differential diagnosis, increase in NF levels and reactive Ab provide a sensitive means for detecting the onset as well as the progression of neuronal degeneration. Fialova et al. used anti-NF Aab profiling to monitor disease progression in patients diagnosed with early MS and clinically isolated syndrome 112. In addition to distinguishing various phases of MS (i.e. relapsing-remitting), the approach showed potential for detecting secondary progressive phases of the pathology related to continuous spillage of NF. It is important to note that, like Aβ, tau, and α-syn Aabs, NF Aabs can be detected in the serum and CSF of not only diseased but also healthy individuals 112, 113 (Table 2). Under certain conditions, NF Aabs seem to contribute to the pathogenesis of several NDDs and can aggravate the disease process in AD patients 114-117.

Soussan et al. compared NF Aab profiles in serum of AD patients and healthy controls. Unlike controls showing equal binding for different isoforms of NF-H (bovine ventral root and dorsal root NF-H) without changing their specificity during aging, in AD, the levels of Aabs against ventral root cholinergic NF-H was higher than those directed against dorsal root NF-H. The phosphoserine content analysis of NFs showed its higher levels in ventral as compared to that of dorsal root NF-H, with Aabs from AD patients binding more effectively phosphorylated epitopes, which show higher prevalence in ventral root NF-H 118. Of note, serum levels of NF-H Aabs in AD patients were lower as compared to healthy controls, while the levels of NF-L Aabs remained unaltered 52. Moreover, AD patients had elevated intrathecal synthesis of tau and NF-H Aabs 79 while patients with multi-infarct dementia showed higher titers of NF-H IgG as compared to the serum of healthy controls. In the context of the current discussion, it is important to note that the prevalence of sub-classes of NF Aabs varies in different neuropsychiatric diseases, which might be due to the immunogenicity of different NF sub-classes (NF-H/NF-M/NF-L) resulting from various modifications of the protein and epitope sites, including phosphorylation of the C-terminal domain of NF, which might impact the pathogenicity of NF Aabs 115, 118.

## α-Synuclein Aabs

Accumulation of insoluble and misfolded α-syn in neurons leads to synaptic failure with the build-up of fibrils constituting Lewy bodies (LB) and neurites of DLB and PD 4, 119. Based on the localization and clinical signs of LB, the Newcastle-McKeith criteria distinguishes three main forms: (1) brain stem predominant form, affecting IX-X motor nucleus, locus coeruleus, and substantia nigra (2) limbic form affecting the amygdala, trans-entorhinal cortex, and cingulate cortex, and (3) neocortical form targeting frontal, temporal and parietal areas 120, 121. Considerable data suggest that α-syn upregulation alone can lead to synaptic pathology and set on the formation of LB, even with retained physiological conformation 122. Like Aβ and tau protein, pathological increase in α-syn is associated with local immune reaction in the brain as well as systemic response. In the PD brain, for instance, aggregates of α-syn in substantia nigra co-localize with deposits of IgG 123, indicating that α-syn build-up induces local Aab response. Of note, exogenously applied monoclonal antibodies to α-syn can alter the rate of protein aggregates in cellular models and animal studies of PD 124-126, inferring that α-syn Aabs may influence the onset and progression of the disease 127, 128.

The results of the analysis of α-syn Aabs in the blood of PD patients and comparison with controls vary considerably (Table 3). While some reports found α-syn Aabs titers unaltered 129-131, others showed significant changes. Besong-Agbo and co-workers 132, for instance, report lower α-syn Aabs levels in sera of PD compared to healthy controls and AD patients. Another small cohort study divided patients into two groups (1) with ≤5-years and (2) ≥10-years PD and described higher levels of α-syn Aabs in sera of both PD patient groups compared to healthy controls. Interestingly, the antibody activity in the second group of patients gradually declined over time, implying that the auto-immune response can be regulated throughout the disease process 128. Similar findings were reported by other studies of α-syn Aabs in PD sera 133 and plasma 134. The level of α‐syn Aabs and changes appears to be gender-dependent, with PD and healthy men showing typically higher titers than women 135. In addition to the blood, alterations of α-syn were investigated in the CSF. Akhtar, Horvath et al 134, 135 have found higher CSF Aabs levels in PD, unlike Heinzel et al 129 reporting no differences from healthy controls. α-syn Aabs levels were also reported to be increased in DLB, and to a lesser extent in AD 136, 137 (Table 3). Finally, a significant rise of α-syn Aabs was found in behavioral variant FTD (bvFTD) patients, where serum levels of α-syn Aabs were significantly higher compared to PD patients 91. Overall, from the autoimmune point of view, the response of Aabs to α-syn varies widely across several NDDs and can be influenced by multiple factors, including the stage of diseases, its severity, patient gender, and others.

## Conclusion and future directions

Despite two decades of in-depth research and major progress in developing biomarkers for CNS disorders, the definitive diagnosis of NDDs remains a major challenge. The current diagnostic gold standard - positron emission tomography (PET) - has low sensitivity and is of limited availability, due to high costs and requirements for specialized infrastructure and skilled staff, as well as potential health risks related to the use of radioactive tracers. Substantial drawbacks are also associated with the use of CNS tissue as well as CSF-based assays involving biopsies and lumbar puncture, which necessitates invasive procedures and related major health risks. The emerging Aabs based blood tests seem to offer a specific, rapid, and affordable approach for diagnosis of NDD without major risks and adverse effects. Nevertheless, significant challenges and questions remain, which impede their effective translation and widespread clinical use, calling for further research and optimization in clinical trials. One of the key difficulties is imposed by the discovery of significant amounts of neuronal Aabs in the peripheral circulation in healthy subjects, inferring their potential physiological role, and questing the specificity of selected Aabs for a particular NDD. Another major challenge is imposed by the results of comparative studies, which demonstrate considerable variations of Aabs levels in the peripheral circulation of NDDs that frequently deviate from changes in Aabs titers in CSF. These observations also substantiate the highly complex nature of the immune response to NDDs and underscore the potential shortcomings of utilized detection methods. Together with numerous conflicting reports and outstanding methodological issues, the above-listed considerations call for revision and improvements of sample preparation and standardization of sensing methods. They also highlight the need for more stringent stratification of target groups and profiling of Aabs, to ensure accurate and specific detection and quantification of Aabs. In this context, the use of genetic methods is especially warranted, given the causative and predisposing effects of specific genes in NDDs. Because of the association of NDD with genetic alterations (e.g., AopE4) 138, 139, the latter might also influence the level and activity of Aabs as biomarkers. Importantly, the emerging inconsistent data highlight numerous outstanding biological questions, which require careful analysis and interpretation. For instance, the cellular origin, induction mechanisms, and potential significance of the physiological presence of Aabs in peripheral circulation remain to be elucidated. Likewise, it must be shown if higher levels of oligomer Aabs as compared to monomers, and changes in their ratio, is of any diagnostic or biological importance under normal and disease conditions. Finally, major outstanding questions remain in the basic neurobiology of neurodegenerative diseases, with important, previously unknown, mechanisms regulating the production, processing, and secretion of amyloid peptides reported recently 140, 141. As binding of Aabs can influence the propensity of α-syn, tau, or Aβ42 for aggregation in fibrillary deposits, alterations in titers of oligomer-specific Aabs might influence the onset of amyloid depositions as well as the pathological spread of misfolded proteins throughout the CNS. Whether this is the case or not remains to be demonstrated. Addressing these and many other technical challenges and scientific questions underscored throughout this study warrants further preclinical research and clinical trials, with the view of improving the diagnostic and therapeutic utility of Aabs in the foreseeable future.

## Figures and Tables

**Figure 2:**
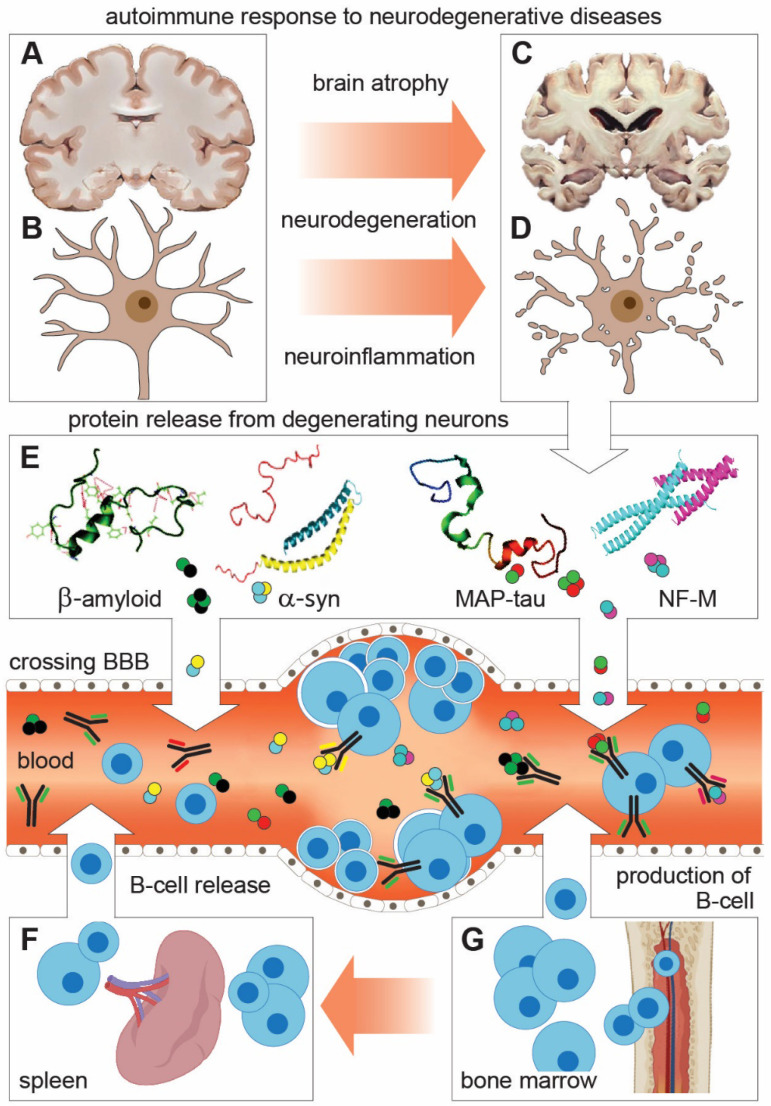
** Schematic representation of shared features and mechanisms of autoimmune response to neuronal proteins in neurodegenerative diseases (NDDs). (A-D)** Degeneration of neurons leads to brain atrophy and the release of neuronal proteins and their fragments (autoantigens) in the interstitial space and CSF. **(E)** From there, autoantigens cross BBB to enter the peripheral circulation (Blood and lymphatic system) where they encounter and activate B lymphocytes to produce and release neuronal protein-specific autoantibodies (Aabs). This reaction underlies changes in Aabs profile and activity in peripheral circulation, which can be taken as indicative (biomarker) for NDDs. **(F and G)** B lymphocytes are produced in the bone marrow to be released directly in blood or stored in the spleen and released in blood as part of the immunogenic response.

**Table 1:** A summary table of Aβ Aabs values in NDD patients versus controls

Directionality	Index change	Diagnosis	Material	Method	Aβ, variant	Reference
Increase	1.33; 1.41	AD	Serum	ELISA	Mono- Agg-	63
No change	1.04	AD	Serum	ELISA	-	58
Decrease	0.53	AD	Serum	IP	-	67
Decrease	0.69	AD	CSF	ELISA	-	59
Increase	10.2; 47.5	AD<5y; AD>15y	Serum	ELISA	Oligo-	152
Increase	40; 5; 160; 30	AD short, long (stages)	Serum	ELISA	-	70
Increase	1.05 - 1.27	AD, mild, severe	Serum	ELISA	Mono- Oligo-	61
Increase	2.2	AD	Serum	ELISA	-	66
No change	0.95	AD	Plasma	ELISA	-	60
Decrease	0.41	AD	Serum	ELISA	-	65
Increase	1.23	AD	Serum	ELISA	-	74
Decrease	0.71	AD	Serum	ELISA	Oligo-	68
Decrease	0.69	AD	Serum	ELISA	-	153
Decrease	0.51	AD	Serum	ELISA	-	69
No change	1.0	AD	Plasma	TAPIR	-	154
No change	1.0	AD	Plasma	Pep. microarray	Oligo -	48
Decrease	0.88	AD	Serum	ELISA	-	155
Increase	1.36-1.69	AD	Serum	ELISA	-	66, 72
No change,	0.96; 1.0	PD, PDND	Serum, CSF	ELISA	-	137, 156
Increase	3.68	AD	Plasma	EIA/RIA	-	62
Decrease	0.63	AD	Serum	ELISA	Fragments	64
No change	1.01	VD	CSF	ELISA	-	137
Increase	1.35; 1.14	DLB/PD; AD/FTD	CSF	ELISA	-	137

**Table 2:** A summary table of tau and neurofilament Aabs values in NDD patients versus controls

Directionality	Index change	Diagnosis	Material	Method	MAP tau, variants	Authors/Year
No change	0.83, 1.0	MCI, AD	Serum	ELISA	IgM non p- tau	80
No change	0.95, 0.66	MCI, AD	Serum	ELISA	IgM p-tau	80
Increase	1.7, 1.02	MCI, AD	Serum	ELISA	IgG p-tau	80
Increase	1.7	AD	Intrathec. synth.	ELISA	-	79
Decrease	0.80	AD	Serum	ELISA	-	52
Decrease	0.45, 0.68	MCI	Serum, CSF	ELISA	-	157
Increase	2.0	AD	Serum	ELISA	p-tau	83
Increase	2.5	MS	Intrathec. synth.	ELISA	-	158
Increase	1.95	PD vs PDND	Serum	ELISA	-	91
Increase	2.2	AD	Intrathec. synth.	ELISA	NF-H	79
Decrease	0.62	AD	Serum	ELISA	NF-H	52
No change	1	AD	Intrathec. synth.	ELISA	NF-L	79
No change	1	AD	Serum	ELISA	NF-L	52

**Table 3:** A summary table of α-synuclein Aabs values in NDD patients versus controls

Directionality	Index change	Diagnosis	Material	Method	α-syn variant	Authors/Year
Decrease	0.90, 0.91	VD, AD/FTD	CSF	ELISA	-	137
Increase	1.27	DLB/PD	CSF	ELISA	-	137
Increase	1.53	PD	CSF	ELISA	-	135
Decrease	0.94, 0.69	AD, PD	Serum	ELISA	-	132
No change	0.63	PD	Serum	ELISA	-	130
No change	0.61, 0.81	PD	Serum, CSF	ELISA	-	129
No change	0.82	PD	Serum	ELISA	-	131
Increase	16.2, 4.0;4.0, 2.0	PD<5y PD>10y	Serum	ELISA	Mono- Oligo, -	159
Increase	1.39, 1.3;1.29, 1.2	PD mild,moderate	Serum,CSF	ELISA	-	134
No change	1.1, 0.9	PD, PDND	Serum, CSF	ELISA	-	129, 137
Increase	1.3 - 3.7	PD	Serum	EIS	-	160
Increase	2.5 - 6.0	PD	Serum	ELISA	Mono-	133
Conditional	-	PD	Serum	WB	-	161
Decrease in HA Abs	1.37	PD	Plasma	ELISA	-	162
Increase	2.5	PD	Serum	ELISA	-	163
Increase	6.3 - 10.7	PD	Serum	ELISA	-	164
Increase	1.32	PD	Serum	EIS	-	165
No change	1	PD, PDND	Serum	ELISA	-	91
